# Pathogenesis and Management of COVID-19

**DOI:** 10.3390/jox11020006

**Published:** 2021-05-21

**Authors:** Khalid O. Alfarouk, Sari T. S. AlHoufie, Samrein B. M. Ahmed, Mona Shabana, Ahmed Ahmed, Saad S. Alqahtani, Ali S. Alqahtani, Ali M. Alqahtani, AbdelRahman M. Ramadan, Mohamed E. Ahmed, Heyam S. Ali, Adil Bashir, Jesus Devesa, Rosa A. Cardone, Muntaser E. Ibrahim, Laurent Schwartz, Stephan J. Reshkin

**Affiliations:** 1 Hala Alfarouk Cancer Center, Department of Evolutionary Pharmacology and Tumor Metabolism, Khartoum 11123, Sudan; derma55@yahoo.com; 2 Research Center, Zamzam University College, Khartoum 11123, Sudan; rector@zamzam.edu.sd; 3 Medical Laboratories Technology Department, College of Applied Medical Sciences, Taibah University, Medina 42353, Saudi Arabia; shoufie@taibahu.edu.sa; 4 College of Medicine, University of Sharjah, Sharjah 27272, United Arab Emirates; samahmed@sharjah.ac.ae; 5 Pharmacology Department, Faculty of Medicine, Fayoum University, Fayoum 63514, Egypt; mfs01@fayoum.edu.eg; 6 Department of Oesphogastric and General Surgery, University Hospitals of Leicester, Leicester LE5 4PW, UK; Ahmed.ahmed@doctors.org.uk; 7 Pharmacy Practice Research Unit, Clinical Pharmacy Department, College of Pharmacy, Jazan University, Jazan 45142, Saudi Arabia; ssalqahtani@jazanu.edu.sa; 8 Department of Medical Laboratories Sciences, College of Applied Medical Sciences, Najran University, Najran 66446, Saudi Arabia; asmezher@nu.edu.sa; 9 Department of Pharmacology, College of Pharmacy, King Khalid University, Abha 61421, Saudi Arabia; amsfr@kku.edu.sa; 10 Department of Preventive Dental Sciences, Ibn Sina National College, Jeddah 22421, Saudi Arabia; amramadan@ibnsina.edu.sa; 11 Department of Surgery, Faculty of Medicine Al-Neelain University, Khartoum 11111, Sudan; 12 Faculty of Pharmacy, University of Khartoum, P. O. Box 321, Khartoum 11111, Sudan; Heyam57@hotmail.com; 13 Institute of Endemic Diseases, University of Khartoum, Khartoum 11111, Sudan; mibrahim@iend.org; 14 Scientific Direction, Foltra Medical Centre, 15886 Teo, Spain; jesus.devesa@usc.es; 15 Department of Biosciences, Biotechnologies, and Biopharmaceutics, University of Bari, 70126 Bari, Italy; rosaangela.cardone@uniba.it (R.A.C.); stephanjoel.reshkin@uniba.it (S.J.R.); 16 Assistance Publique des Hôpitaux de Paris, 75003 Paris, France; dr.Laurentschwartz@gmail.com

**Keywords:** COVID-19, SARS-COV-2, virus, cytokine storm, pharmacology

## Abstract

COVID-19, occurring due to SARS-COV-2 infection, is the most recent pandemic disease that has led to three million deaths at the time of writing. A great deal of effort has been directed towards altering the virus trajectory and/or managing the interactions of the virus with its subsequent targets in the human body; these interactions can lead to a chain reaction-like state manifested by a cytokine storm and progress to multiple organ failure. During cytokine storms the ratio of pro-inflammatory to anti-inflammatory mediators is generally increased, which contributes to the instigation of hyper-inflammation and confers advantages to the virus. Because cytokine expression patterns fluctuate from one person to another and even within the same person from one time to another, we suggest a road map of COVID-19 management using an individual approach instead of focusing on the blockbuster process (one treatment for most people, if not all). Here, we highlight the biology of the virus, study the interaction between the virus and humans, and present potential pharmacological and non-pharmacological modulators that might contribute to the global war against SARS-COV-2. We suggest an algorithmic roadmap to manage COVID-19.

## 1. Introduction

Deaths due to SARS-COV-2 infection have officially surpassed 3 million, with the probable number of victims being much higher. The latest surges of contagion are perhaps stronger than the original wave and seem to be unstoppable. While vaccination is considered the best solution to eradicating the virus and halting the pandemic, the number of people vaccinated worldwide is very small, especially in the nations currently hardest hit, and probably will proceed very slowly. This leaves the health services that must save the lives of people already infected with few options at their disposal. The need is particularly critical given that the available evidence suggests the current treatment modalities have only a minimal impact on survival. Indeed, the significant variability in the clinical picture and outcomes in COVID-19 patients means that any single, comprehensive approach to treatment is a dead-end road. Further, our perceptions of COVID-19 and what we know of the virus, diagnosis, disease symptoms, course, treatment, and aftermath of its infection have changed with time.

If patients are to be treated with new possible therapeutic options, the basic biology of the infection process, especially the proteins and physiological conditions associated with the phases of the development of the disease, must be delineated to understand the challenges and tailor treatment to each individual. In this review, we highlight the biology of the virus, discuss the interaction between the virus and humans, and present an algorithmic roadmap of potential pharmacological and non-pharmacological modulators that might reduce the clinical effects of SARS-COV-2 infection. Adopting individualized medicine and pharmacodiagnostics might represent a more effective rationale, at least in severely ill patients.

## 2. Historical Preface

**Figure 1:**
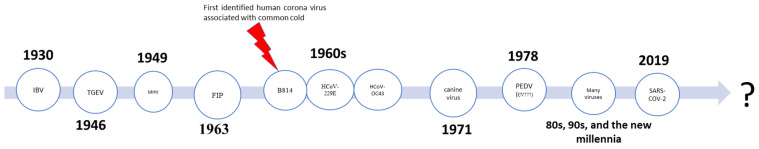
Summary of the timeline of discovery of many of coronavirus family members.

In 1930, a newly isolated virus strain caused acute respiratory infection in chickens, termed infectious bronchitis virus (IBV) [1,2]. Then, two additional animal viruses were isolated: In 1946, transmissible gastroenteritis virus (TGEV) affected pigs, and in 1949, mouse hepatitis virus (MHV) affected mice [3,4]. In the 1960s, the era of human coronaviruses started when David Tyrrell and ML Bynoe isolated a viral strain, called virus B814, from respiratory samples from a schoolboy with a common cold [5,6]. In the same decade, two additional species were isolated including human coronavirus 229E and human coronavirus organ culture 43 (HCoV-OC43) [7,8]. Almeida et al. called this group of viruses “coronavirus” as they resemble the “solar corona” or they are “in a crown” [9]. Feline infectious peritonitis virus in was isolated in 1963 [10], canine virus was isolated in 1971 [11,12], and porcine epidemic diarrhea virus (PEDV) was isolated in 1978 [13,14]. Finally, at the end of December 2019, three patients from the seafood and wet animal wholesale market in Wuhan, China were admitted to the hospital with pneumonia of unknown causes [15]. Later, Zhu et al. reported a novel beta-coronavirus, “CoV (2019-nCoV),” as the cause of this pneumonia. This novel virus was named “novel coronavirus-infected pneumonia” (NCIP), later known as SARS-COV-2, [15], and has evolved, consequently, into ten subtypes thus far [16] (See Figure 1).

## 3. Virus Biology

The entry process depends on several factors, including virus-related factors (e.g., binding proteins) and host-related factors (e.g., tissue tropism or the tissue’s ability to receive and accommodate the virus) [17]. The process of virus fusion and entry is more complex, but, in general, it depends on acidification and/or proteolytic activation [18,19]; therefore, it is not surprising if systemic alkalization would be a rational approach to disrupt virus entry [20]. The fusion process occurs between the interaction of virus proteins and host receptor proteins. There are many viral proteins, one example being the S protein. The S protein is one of four structural proteins encoded by the CoV single-stranded, positive-sense RNA genome. In the viral membrane, the S protein functions to (i) interact with the cellular receptor and (ii) induce viral fusion with the cell via the plasma membrane protein angiotensin-converting enzyme 2 (ACE2) [21,22,23,24].

The S protein needs to be primed by an appropriate protease at the S1 and S2 interface (S1/S2) to catalyze the membrane fusion reaction and trigger the FPs immediately upstream (S2′). What is interesting about this triggering event is that several proteases can trigger it and it is the protease requirements that drive viral tropism.

Both SARS-CoV and MERS-CoV S can be triggered to fuse at either the plasma membrane or the endosomal membrane, and their route of access is determined by protease availability while being governed by the attachment of the surface unit, S1, of the S protein to a cellular receptor, facilitating viral attachment to a target cells’ surface. Additionally, access involves S protein priming by cellular proteases, which involves S protein cleavage at the S1/S2 and the S2′ site and allows fusion of viral and cellular membranes, a process driven by the S2 subunit [25]. The SARS-COV-2 engages ACE2 as the entry receptor and employs the cellular serine protease TMPRSS2 for S protein priming [25].

Angiotensin-converting enzyme 2 (ACE2) receptors were shown to be the SARS-CoV-2 gateway to the cell [20,21,22]. ACE2 is an enzyme affixed to the plasma membrane (outer membrane) and is widely expressed among various human tissues. Expression levels differ from higher expression (small intestine, testis, kidneys, heart, thyroid, and adipose tissue), to medium expression (lungs, colon, liver, bladder, and adrenal gland), and to the lowest expression (blood, spleen, bone marrow, brain, blood vessels, and muscle) [26]. While lungs show medium expression compared to other tissues, SARS-COV-2 initially infects the lung [27]. However, we do not know whether the virus is disseminated to other parts of the body or not [21]. If yes, it will be of significant progress to know whether the multiple organ failure is associated with the side effect of drugs, ARDS, or with virus entry to that tissue [28,29,30,31,32].

**Figure 2:**
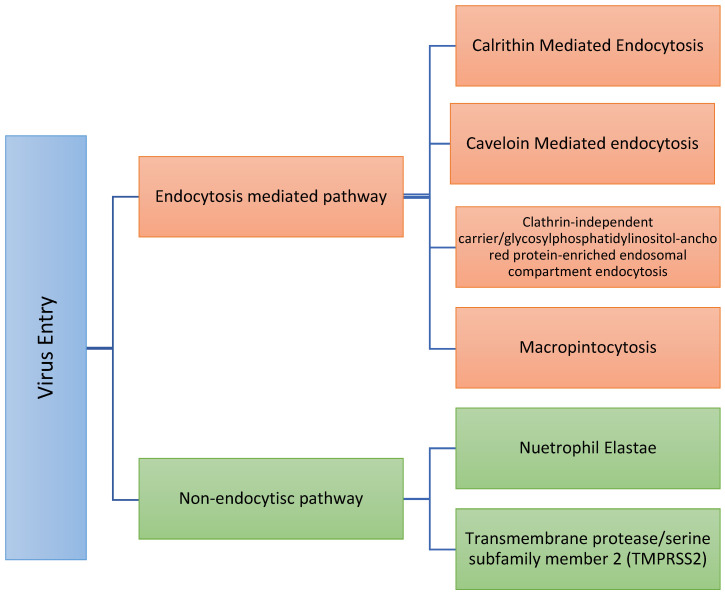
Summary of the different routes of virus entry. Therefore, proper determination of the exact route of entry might enhance the selection rationale for which targeted therapy should be used.

After viral fusion with the plasma membrane, virus internalization can occur through various routes (Figure 2) [33,34,35]:

***Non-endocytic pathway:*** This pathway could also be termed a “proteases-directed internalization pathway.” After S protein binding to the receptor (not before), host proteases mediate virus–cell fusion [21,35,36]. Some of these proteases are trans-plasma membrane proteins. They play a crucial role in COVID-19. Some of these proteases include the following:

- Neutrophil elastase (NE): Elastase is a serine protease that also hydrolyzes amides. Neutrophil elastase is one of eight elastases found in the body. That NE could mediate entry is clinically vital since elastase is produced by neutrophils in the lungs during SARS-CoV-2 infection and could promote the progression of SARS-CoV-2 infection [16]. Therefore, as the hallmark of COVID-19, further neutrophils recruitment supports the release of a massive amount of NE and, subsequently, further virus entry [37,38,39] in a positive feedback cycle. Sivelestat is a NE inhibitor [40,41] and, so, might limit SARS-COV-2 entry. In this respect, we are not sure if the medications that decrease neutrophil counts will represent a potential treatment or not. Some of these medications include Carbimazole, Clozapine, Dapsone, Dipyrone, Methimazole, Penicillin G, Procainamide, Propylthiouracil, Rituximab, Sulfasalazine, and Ticlopidine;
- Type II transmembrane serine proteases, including transmembrane protease/serine subfamily member 2 (TMPRSS2) [42], also known as human epitheliasin [43]. Human TMPRSS2 mRNA is expressed in several tissues, including the prostate, ovary, breast, lung, kidney, pancreas, bile duct, salivary gland, stomach, small intestine, and colon [43,44,45]. Transient expression of TMPRSS2 enhances SARS-CoV-2 S-mediated cell–cell fusion [21]. S protein is primed by TMPRSS2 and, therefore, TMPRSS2 supports virus entry, viral fusion, and spread and further increases the virus [46]. Nafamostat and Camostat are transmembrane protease inhibitors; serine 2 acts as a TMPRSS2 inhibitor [25,47,48,49]. Further, Nafamostast acts as an anticoagulant drug, which might shed light on how the virus induces thrombosis. Camostast is used for the treatment of pancreatitis. TMPRSS2 expression is subjected to population variability, e.g., TMPRSS2 expression is relatively lower in darker skin than in the white populations, suggesting that the probability of TMPRSS2-dependent virus internalization might be higher in a white population [50]. In addition, the expression of TMPRSS2 increases with aging [49], and TMPRSS2 expression is positively correlated with androgen levels, i.e., the male population [50].

Although TMPRSS2 inhibition could be presented as a promising approach in managing SARS-COV-2 [25], we are not sure if blocking of TMPRSS2 will stop the viral infectivity or if the virus will adapt to such inhibition and undergo cellular internalization through another endocytic pathway, because if the plasma membrane-route proteases are available, the virus can fuse via an “early pathway” at the plasma membrane and, if not, the virus can fuse via a “late pathway” at the endosomal membrane [25].

The ***Endocytic pathway*** also could be termed as a receptor-mediated endocytosis pathway. The endocytosis pathway can be carried out through different routes, e.g., clathrin-mediated endocytosis, caveolin-mediated endocytosis, flotillin-dependent endocytosis, macropinocytosis, and clathrin-independent carrier/glycosylphosphatidylinositol-anchored protein-enriched endosomal compartment endocytosis [33,51,52,53,54,55]. An acidic environment activates endosomal proteases such as cathepsin B and cathepsin L, which are known activators of other CoV family members, that become active in the early and late endosomes. To rationally target each approach, it will be wise to determine which route the virus follows to enter the cell endocytotically and then target it as follows.

- Clathrin-mediated endocytosis, the endosomal/lysosomal pathway, is the uptake of material (e.g., ferritin, LDL particles) into the cell from its surface using clathrin-coated vesicles [56], and the clathrin-coated vesicle changes its geometry to accommodate endocytosis [57,58]. Clathrin-mediated endocytosis can be inhibited by Pentamidine [20], dynasore [59], monodansylcadaverine (MDC) [20], depletion of intracellular potassium [20], phenylarside oxide (PAO) [60], cytosolic acidification (ammonium chloride (NH_4_Cl)) [61], hypertonic shock (sucrose) [62], and chlorpromazine [63]. Importantly, in this respect, a group of French scientists observed a lower prevalence of symptomatic and severe forms of COVID-19 infections in psychiatric patients treated with the anti-psychotic drug chlorpromazine, and formulated the hypothesis that chlorpromazine might be a preventive against COVID-19 [20]. Following this observation, a clinical trial has been set-up [64].
- Caveolin-mediated endocytosis: Caveolae were described first in 1953 by Palade [65,66]. Caveolae and caveolin-containing membrane domains on the plasma membrane have various curvatures and shapes [66]. When the clathrin vesicles fuse with endosomes/lysosomes, the caveosome (multi-caveolar complexes) never fuses with lysosomes and, hence [67,68], it will not be surprising if SARS-COV-2 acts more through caveolin-mediated endocytosis [33] as that represents a successful anti-predator strategy. Vanadate, a tyrosine phosphatase inhibitor, stimulates caveolin-mediated endocytosis, while Nystatin (anti-fungal drug) suppresses the caveolin-mediated endocytosis, and chlorpromazine is non-specific [69]. Brefeldin A (antiviral drug) [70] and Nocodazole (anti-neoplastic drug) [71] also inhibit the caveolin pathway [72].
  - Cathepsin B (catB): Cathepsin B is a lysosomal cysteine protease that belongs to the papain family [20,73]. Cathepsin B plays a vital role in intracellular proteolysis. In normal physiological conditions, active cathepsin B is localized to the endosomal/lysosomal compartment and is primarily involved in the normal turnover of intracellular and extracellular proteins, thus maintaining homeostatic metabolic activity within cells [71]. Beyond its effect in the mitochondrial complex I, metformin acts as a catB-inhibitor [73,74]. However, inhibition of catB will result in thyroid dysfunction, as catB is necessary for thyroxin production. Therefore, the targeting of catB should be cautiously monitored.
  - Cathepsin L (catL): Cathepsin L can degrade nearly all proteins, including enzymes, receptors, and transcription factors [75]. The physiological function of catL depends on its subcellular localization as follows:
    o In endosomes/lysosomes: As it degrades the proteins in lysosomes, catL plays a crucial role in maintaining the lysosome–endosome compartment of the cardiac myocyte. Therefore, any alteration of catL might induce a progressive dilated cardiomyopathy [76,77,78,79]. In addition, disruption of catL might decrease CD+ T cells.
    o In the nucleus: Cathepsin L is a double-edged sword, it can either accelerate or inhibit the proliferation based on a couple of factors [75].
    o In the cytoplasm: Cathepsin L initiates the lysosomal pathways of apoptosis [80,81,82].
    o In the extracellular space: In the inflammatory environment, pro-inflammatory cytokines induce catL expression in endothelial cells, macrophages, and smooth muscle, then the released catL degrades elastin and collagen [75] and so might disrupt the matrix.

Cathepsin L has been identified as a highly effectual enzyme to degrade the viruses’ outermost capsid and underlying proteins as a criterion to initiate an infection. The viruses access the endosomal–lysosomal compartment and thereafter disassemble by endocytosis. The subvirion particles penetrate to the cytoplasm to replicate [75,83]. Therefore, catL plays a critical role in the entry of the SARS-COV-2 virus [84]. Cathepsin L inhibitors such as CTLA-2α [85], the selective N-(benzyloxycarbonyl)-L-phenylalanyl-L-tyrosinal [86], MDL28170 [87], and even slight alkalization show a gradual reduction in fusion due to catL activity [87].

## 4. Cytokine Storm

It seems that the first introduction of the term cytokine storm was used in early 1993 to describe the role of interleukin 1 on graft-versus-host disease [88]. Cytokine storm might be a synonym to immune attack or massive immune response against foreign bodies, e.g., bacteria [89] and viruses [90,91,92], where SARS-COV-2 is one of those viruses. However, the cytokine storm in COVID-19 surely does not develop directly as an immune response to the virus [93,94] nor as a result of the multiple organ failure [95] due to COVID-19 [93]. A cytokine profile resembling systemic lupus erythematosus (SLE) and/or secondary hemophagocytic lymphohistiocytosis (sHLH), is associated with COVID-19 disease severity and is characterized by an increase in the following:

Interleukin 1 beta (IL-1β), also termed lymphocyte activating factor, leukocytic pyrogen, leukocytic endogenous mediator, or mononuclear cell factor is a pro-protein converted to mature IL-1β by cytosolic caspase 1 [96] and produced by activated macrophages [97]. IL-1β plays a crucial role in mediating autoimmunity [98]. The monoclonal anti-IL-1β antibody, canakinumab, is a suggested inhibitor [99,100]. In this regard, Novartis started a clinical trial to show the effect of Canakinumab against COVID-19 [101]. Curcumin also shows an inhibitory effect on IL-1 [102].

Granulocyte-colony stimulating factor (G-CSF) is a secreted glycoprotein produced by macrophages, endothelial cells, stromal cells, natural killer cells, and T cells [103]. There are four types of CSF: granulocyte-colony stimulating factor, macrophage-colony stimulating factor, granulocyte-macrophage-colony stimulating factor, and multi-colony stimulating factor (interleukin-3) [104]. G-CSF supports production and differentiation of white blood cells. G-CSF supports neutrophil proliferation, and GM-CSF supports macrophages and eosinophils proliferation [105]. Administration of G-CSF is associated with increased lactate dehydrogenase, uric acid, isoenzymes, alkaline phosphatase, and the increase in soluble IL-2 receptors [104], which are found at higher levels in serum of COVID-19 patients [106,107,108,109]. G-CSF plasma levels were also correlated with the severity of COVID-19 (higher in patients in the intensive care unit) [110,111,112]. Therefore, targeting the G-CSF/G-CSF receptor axis to manage COVID-19 should be considered.

Interferon-gamma-inducible protein-10 (IP-10)/CXCL10, also called IP-10, is a small protein chemokine (10kDa) induced by IFN- γ and produced by many cell types, e.g., neutrophils, monocytes, endothelial cells, fibroblasts, and keratinocytes. Physiologically, CXCL10 recruits natural killer cells (NK cells), eosinophils, and leukocytes and increases the Th1/Th2 ratio [113,114,115]. CXCL10 also increases the level of several inflammatory mediators, including TNF-α that promotes inflammation. Therefore, CXCL10 is considered an inflammatory chemokine that contributes to many autoimmune diseases [113,116,117]. The CXCL levels increase 10-fold in COVID-19 and they play a paramount role in its immunopathology [118,119]. CXCL10 inhibition might represent a rational approach to the target, and its inhibitors include Vitamin D [120], Thiazolidinediones [121], Ganodermycin [120], and artemether combined with atorvastatin [120,122,123], although the earlier data about the last example suggested the reverse effect.

Macrophage inflammatory protein 1-α (MIP 1-α) belongs to the family of chemokines, also known as MIP-1alpha chemokine (C-C motif) ligand 3 (CCL3) [124]. Upon activation, they can be expressed by hematopoietic cells and a variety of tissue cells such as fibroblasts, epithelial cells, vascular smooth muscle cells, or platelets [125,126]. MCP 1-α are best known for their chemotactic and pro-inflammatory effects and are crucial for immune responses towards infection and inflammation and are produced by countless cells, particularly macrophages, dendritic cells, and lymphocytes [127]. MIP 1-α is associated with COVID-19 [128,129] and, therefore, targeting MIP 1-α directly or altering it at the receptor level (CCL3 blocker) represents a wise approach to managing COVID-19 [130]. IL-10 inhibits MIP 1-α expression [131].

Monocyte chemoattractant protein 1(MCP-1/CCL2) is also known as the small inducible cytokine A2. Although monocyte/macrophages are the primary sources of CCL2, many cells also produce MCP1, such as endothelial, epithelial, smooth muscle, fibroblasts, mesangial, astrocytic, monocytic, and microglial cells [132,133,134,135]. Those cells are responsible for antiviral immunity. Many factors stimulate MCP-1 production, such as oxidative stress, growth factors, and cytokines. MCP-1 is the critical regulator of migration and infiltration of monocytes and macrophages [132]. MCP1 recruits monocytes, dendritic cells, and T-cells (memory) to the inflammation site because of inflammation and infection [136]. MCP-1 is subject to population variability as it is higher in whiter skin than in darker skin populations [137,138]. MCP-1 is associated with COVID-19 [139,140,141]; therefore, targeting MCP-1 could also be considered in managing SARS-2 infection [130]. Melatonin inhibits MCP-1 expression [142]. Bindarit decreases MCP-1 synthesis [143]. Spiegelmer (L-enantiomeric RNA oligonucleotide mNOX-E36), also called L-ribonucleic acid aptamer, inhibits MCP-1 activity [144]. Furthermore, MCP-1 could be blocked at the receptor level (CCR2 blockage) [143] using compounds such as 747 (a natural combination related in structure to kaempferol) [145] and 15a (an orthostatic CCR2, a small molecule antagonist) [146].

Tumor necrosis factor-α (TNF-α) is also called cachexin or cachectin. TNF-α is a cell-signaling protein produced mainly by macrophages in response to stimuli and mediates the inflammatory response [147,148,149]. A great deal of data showed that TNF-α has a receptor association with COVID-19 and higher TNF-α expression is correlated with disease severity and higher mortality [150,151,152,153]. It is not surprising that anti-TNF-α drugs (adalimumab (A), certolizumab pegol (C), etanercept (E), golimumab (G), and infliximab (I)) are considered anti-COVID-19 treatments [152,153,154,155,156,157] (Table 1).

## 5. Notes on Some Currently Administered Pharmacological Modulating Agents

Administration off-label of certain drugs (drug repurposing) that target the virus and/or the cytokine storm has become a promising approach in managing COVID-19 [158]; some of these agents include:

Macrolide antibiotics: These antibiotics inhibit bacterial protein synthesis. Therefore, they are clinically used to fight infections by atypical bacterial (bacteria that lack cell walls). Evidence shows their promising activity in the management of COVID-19 [159].

-Azithromycin exerts its immunomodulatory role via the following:

(i) Suppression of LPS-induced MDC and IP-10 expression through the MAPK–JNK and the NFκB–p65 pathways [160];
(ii) Inhibition of the cytoplasmic phospholipase A_2_, so it might be equivalent to steroids that suppress the release of eicosanoids (prostaglandins, thromboxane, leukotrienes, and HEPTE) [161].

-Clarithromycin is another macrolide antibiotic that is useful in the management of COVID-19 due to its following properties:

- Influence on two steps in the influenza virus entry process. The drug was found to reduce the expression of sialic acid residues on the surface of airway epithelial cells, reduce virus binding, and reduce the number of acidic endosomes in the cell, inhibiting endosomal escape [34];
- Alteration of endosomal pH. Clarithromycin also inhibits the release of NF-κB (nuclear factor kappa-light-chain-enhancer of activated B cells) [162] and might support the mortality rate due to SARS-COV-2 [163]. The massive release of cytokines in such conditions raises another possible question: Does the cytokine storm support the viral entry and dissemination across the body or is the cytokine storm just a consequence of massive uncontrolled cell death?
- Stabilization of the mast cells [164]. Mast cells secret both histamine and heparin. Therefore, clarithromycin might prevent the massive release of histamine and prevent anaphylaxis and perhaps the blood coagulation that might result from activation of factor XII through the bradykinin activation. However, the prevailing medical dogma is that heparin is an anticoagulant.

Although macrolides have proven useful in their administration as anti-COVID-19 drugs by attenuating the cytokine storm, they exert additional benefits in the management of patients with COVID-19 by reducing the incidence of bacterial co-infection [165].

Amiloride: Amiloride is a well-known potassium-sparing diuretic. It has been shown to inhibit micropinocytosis [69], which might alter this viral entry pathway. It also inhibits the E protein, which is crucial for viral replication [166].

Sodium bicarbonate: Spike proteins of SARS-COV-2 become more disordered at alkaline pH values while becoming more well-formed at more acidic pH values. Therefore, increasing extracellular pH most probably alters the ACE2–SARS-COV-2 interaction dynamics [167]. Hence, sodium bicarbonate used as a buffer has been suggested as it attenuates the hyper-inflammatory environment [168]. In this respect, potassium citrate as a buffer might also be beneficial in COVID-19 treatment [169].

## 6. Food Supplements That Might Prevent COVID-19 Complications

There are some food supplements that might prevent or decrease the severity of COVID-19 infection; some of these food supplements include:

N-Acetyl Cysteine (NAC): NAC is a precursor of glutathione used to treat paracetamol overdoses and is a mucolytic (loosens thick mucus in the lungs). NAC is an immune booster and an anti-inflammatory and antiviral agent. Therefore, it is a suitable agent for COVD-19 [170], significantly reducing the cytokine storm [171]. NAC has also been used in the management of critically ill septic patients [170].

Vitamin E has a lysosomal membrane stabilization function [172,173] and has an inhibitory effect on the production of several pro-inflammatory cytokines, including IL-1, IL-6, TNF, and the chemokine IL-8, by monocytes and macrophages. Vitamin E can also stimulate the intracellular type I IFN system, which exercises antiviral activities [174]. Therefore, it might be beneficial in prevention and/or management of COVID-19 complications.

Desferrioxamine is an iron-chelating agent. A higher level of transferrin indicates a higher level of iron, which is toxic in humans. Blocking receptor-mediated endocytosis reduced the internalization of some infectious viruses similarly to the reduction of endocytosis by transferrin [175], suggesting that transferrin might be an indicator of how viruses enter the cells and how some possible inhibitors could function by inducing paralysis of receptor-mediated endocytosis, e.g., amiloride and NH4Cl [176]. In this respect, desferrioxamine might show potential activity as an anti-COVID-19 treatment [177].

Zinc is an essential trace element found in humans, plants, and microorganisms. Nearly 10% of human proteins (enzymes, transcriptional factors, etc.) bind with zinc. In plasma, 60% of zinc is bound with albumin and around 10% with transferrin (iron transporter, see above). Thus, if iron is increased, free zinc is reduced (hypozincemia) [178,179]. In COVID-19, the level of iron is increased significantly, and, therefore, the level of free zinc is reduced, and this is associated with poor outcomes [180,181,182]. Thus, a zinc supplement is a wise choice in the management of COVID-19 [183].

Vitamin C is a known antioxidant and its role in treating sepsis has been reported [184,185], which supports the notion that vitamin C could be considered in COVID-19 treatment [186]. A clinical trial involving 140 patients will be conducted in Wuhan, China. The scientists will be assessing the need for the ventilator, vasopressors, organ failure, the length of ICU stay, and the mortality in response to high doses of vitamin C [187].

Vitamin K With the increasing evidence that microthrombi formation is linked to COVID 19 infection [188,189], low molecular weight heparin, Fondaparinux, oral anticoagulants, or vitamin K antagonists have been recommended to all patients with an active infection unless they have a relevant contraindication [190,191,192,193], therefore, the level of vitamin K should be closely adjusted.

Vitamin D (calcitriol), although it is one of the fat-soluble vitamins, it is considered to be a hormone [194,195]. As a hormone, vitamin D activates innate immunity and suppresses adaptive immunity [196,197]. Vitamin D deficiency is also associated with viral infection and is accompanied by acute respiratory distress syndrome [197,198]. Therefore, many trials have been carried to test it against COVID-19.

Melatonin is a hormone mainly secreted from the pineal gland [199]. Melatonin is an enormously powerful antioxidant hormone. Melatonin inhibits the activation of the primary inflammasome NLRP3, which leads to cytokine storms [200]. Besides its activity in the antioxidant cascade [201,202,203,204], melatonin supports the expression of many antioxidant cellular enzymes, including glutathione reductase, glutathione peroxidase, superoxide dismutase, and catalase [205,206]. As some of these enzymes are mitochondrial enzymes, melatonin is also a mitochondrial antioxidant [207,208] and, thus, it can be used to manage COVID-19 [209,210].

## 7. Roadmap to Manage COVID-19 and Concluding Remarks

COVID-19 is an emerging pandemic disease threatening human life. COVID-19 manifests as hyper-inflammation and heterogenous increases or decreases in cytokines, chemokines, electrolytes imbalance, etc. The significant variability in the clinical picture and outcomes in COVID-19 patients means that any blockbuster approach to treatment is a dead-end road. Adopting individualized medicine and pharmacodiagnostics (predictive medicine) might represent a more effective rationale, at least in severely ill patients [211,212].

Prevention by implementing facemask usage is important; some public health practitioners and theoreticians are raising the possibility of herd immunity, but that comes at a high cost of more deaths [213]. This roadmap for optimizing decision making to tailor and fine-tune therapy to the characteristics of the disease and specific needs of the patient includes (i) managing the infection via its signs and symptoms (e.g., cytokine, hyper-inflammation) and prevention of co-infection by targeting the virus and/or the cytokine storm to avoid co-infection, whether bacterial, viral, or by other microorganisms; and (ii) delaying the post-infection toxicity, e.g., thromboembolism. Moreover, the administration of pharmacological and/or nutraceutical compounds that support the repairing of the injured lung epithelium, especially among older people, will represent a promising strategy in the war against COVID-19.

## Figures and Tables

**Table 1:** Cytokines/chemokines that mediate the cytokine storm, with their antagonists.

The Cytokine/Chemokine	Possible Antagonist
Interleukin 1 beta (IL-1β)	Canakinumab [99,100]
Interferon-γ inducible protein 10 (CXCL10)	Vitamin D, Thiazolidinediones, Ganodermycin [120,121]
Monocyte chemoattractant protein 1(MCP-1/CCL2)	Bindarit, Spiegelmer, compounds such as 747, 15a [143,144,145,146]
Macrophage inflammatory protein 1-α (MIP 1-α)	CCL3 blocker, IL-10 [130,131]
Tumor necrosis factor-α (TNF-α)	Adalimumab (A), certolizumab pegol (C), etanercept (E), golimumab (G), and infliximab (I) [154]
